# Expression of CD44 and the survival in glioma: a meta-analysis

**DOI:** 10.1042/BSR20200520

**Published:** 2020-04-15

**Authors:** Gang Wu, Xinghui Song, Jun Liu, Shize Li, Weiqin Gao, Mingxing Qiu, Changjin Yang, Yiming Ma, Yuhong Chen

**Affiliations:** 1 Department of Neurosurgery, Liuzhou People’s Hospital, Liuzhou 545006, China; 2 Department of Rheumatism and Immunology, The Fourth Affiliated Hospital of Guangxi Medical University, Liuzhou 545005, China; 3 Department of Vascular Surgery, Liuzhou People’s Hospital, Liuzhou 545006, China

**Keywords:** CD44, Glioblastoma, Glioma, Meta-analysis, Survival

## Abstract

***Background:*** Higher tumor expression of CD44, a marker of cancer stem cells (CSCs), is associated with poor overall survival (OS) in various cancers. However, the association between CD44 and poor OS remains inconsistent in glioma. We aimed to evaluate the potential predictive role of CD44 for prognosis of glioma patients in a meta-analysis.

***Methods:*** Observational studies comparing OS of glioma patients according to the level of CD44 were identified through searching PubMed, Embase, and Cochrane’s Library databases. Meta-analyses were performed with a random- or fixed-effect model according to the heterogeneity. Subgroup analyses were performed to evaluate the influences of study characteristics.

***Results:*** Eleven retrospective cohort studies were included. Results showed that increased CD44 expression in tumor predicted poor OS in glioma patients (hazard ratio [HR]: 1.42, 95% confidence interval [CI]: 1.02–1.97, *P*=0.04). Subgroup analyses showed that higher tumor CD44 expression significantly predicted poor OS in patients with World Health Organization (WHO) stages II–III glioma (HR: 2.99, 95% CI: 1.53–5.89, *P*=0.002), but not in patients with glioblastoma (HR: 1.26, 95% CI: 0.76–2.08, *P*=0.47; *P* for subgroup difference = 0.03). Results were not statistically different between subgroups according to patient ethnicity, sample size, CD44 detection method, CD44 cutoff, HR estimation, univariate or multivariate analysis, or median follow-up durations (*P*-values for subgroup difference all >0.10).

***Conclusion:*** Higher tumor expression of CD44 may predict poor survival in patients with glioma, particularly in those with WHO stage II–III glioma.

## Introduction

Glioma is a common malignant tumor of the central nervous system. According to previous studies, glioma accounts for 50–80% of brain tumors [1]. Currently, glioma can be staged to I–IV in accordance with the World Health Organization (WHO) 2016 classification, and higher stages indicate increased malignancies [2,3]. Among which, the WHO stage IV glioma, also known as glioblastoma, is the most malignant glioma. The median survival of patients with glioblastoma is <15 months [4,5]. Clinically, the WHO stage system is commonly used for prognostic prediction in glioma patients. However, it has been suggested that prognostic prediction in glioma patients may be complicated, and the prognosis in patients with same WHO stage glioma can vary dramatically [6,7]. Therefore, uncovering novel prognostic factors remains important for improving the quality of care for glioma patients.

Cancer stem cells (CSCs) refer to a small proportion of cancer cells which have the capacities of self-renewal and multidifferentiation [8,9]. Current evidence indicates that CSCs play important roles in tumor progression, metastasis, recurrence, resistance to chemotherapy or radiotherapy, and an overall poor prognosis [10,11]. Previous studies showed that CSCs are characterized by higher expressions of certain molecules, which are collectively termed as CSC marker molecules [12,13]. Increasing evidence indicates that CD44 family is one of the most common CSC markers. Pathophysiologically, CD44 is involved in the processes of cell growth, survival, differentiation, motility, tumor growth, proliferation, and metastasis [14]. Moreover, higher expression of tumor CD44 has been associated with poor prognosis in patients with various cancers, such as non-small cell lung cancer [15], gastric cancer [16], hepatocellular carcinoma [17], colorectal cancer [18], renal cell carcinoma [19], breast cancer [20], ovarian cancer [21], head and neck cancer [22], and osteosarcoma [23]. However, the association between higher expression of CD44 and the overall survival (OS) in patients with glioma showed inconsistent results [24]. Some studies demonstrated that higher tumor expression of CD44 was associated with poor OS in patients with glioma [25–27], while others did not show a significant association [28–34]. Moreover, one of the studies even showed that higher tumor expression of CD44 may be associated with improved OS in glioma patients [35]. Since the sample sizes of such studies are limited, they may be too underpowered to show a significant association between tumor CD44 levels and the prognosis in glioma patients. Therefore, we performed a meta-analysis to quantitatively summarize the predictive power of tumor CD44 expression for OS in patients with glioma.

## Methods

This meta-analysis was designed, performed, and reported in accordance with the Meta-analysis of Observational Studies in Epidemiology [36] and Cochrane’s Handbook [37] guidelines.

### Literature search

The PubMed, Embase, and the Cochrane’s Library electronic databases were queried for relevant studies using the terms ‘CD44’, combined with ‘glioma’, ‘glioblastoma’, ‘glial cell tumor’, or ‘astrocytoma’ from inception to 26 December 2019. The search was limited to human studies published in English or Chinese. The references of the original and review articles were also manually analyzed for possible studies.

### Inclusion and exclusion criteria

Studies were included if the following criteria were fulfilled: (1) full-length article in English or Chinese; (2) included patients with histopathologically diagnosed glioma; (3) observed the association between tumor CD44 expression and OS of the patients;reported hazard ratios (HRs) and 95% confidence intervals (CIs) for poor OS during a follow-up in patients with higher vs. lower CD44 level in tumor, or the data were efficient to estimate the HR and 95% CIs from the survival analysis. Reviews, preclinical studies, and duplicate reports were excluded.

### Data extraction and quality assessment

Database search, data extraction, and quality evaluation were independently performed by two authors. Discrepancies were resolved by discussion with the corresponding author. Data containing the study characteristics (name of the first author, study design, publication year, and location), patient characteristics (age, gender of the patients, and WHO grade of glioma), CD44 measurements (methods and cut-off values), characteristics of survival analysis (HR reported in the context or estimated via the survival curve, univariate or multivariate analyses), median follow-up durations, and variables adjusted for when presenting the results were extracted. Some of the published articles did not provide HR and 95% CI directly. In such cases, two reviewers independently digitized and extracted the data through the Kaplan–Meier survival curves using GetData Graph Digitizer 2.24 (http://getdata-graph-digitizer.com), and then reconstructed the HR and its variance (GraphPad Software, Inc.). We assessed the quality of the included studies using the Newcastle–Ottawa Scale [38], which judges each study according to three domains: selection of the study groups; the comparability of the groups, and the ascertainment of the primary outcome.

### Statistical analyses

Meta-analyses were performed to summarize the association between tumor miR-210 expression and the risk of poor OS outcome. HR and the corresponding standard errors (SEs) were estimated from 95% CIs or *P*-values, and were logarithmically transformed to obtain a normal distribution [37]. The Cochrane’s Q test and *I^2^* test were performed to evaluate the heterogeneity [39]; an *I^2^* > 50% indicates significant heterogeneity. A random-effect model was applied if significant heterogeneity was detected, otherwise, a fixed-effect model was applied [37]. A predefined subgroup analysis was performed to evaluate the influence of study characteristics on the outcome, including patient ethnicity, sample size, WHO grade, CD44 detection method, CD44 cutoff, HR estimation, univariate or multivariate analysis, and median follow-up durations. Medians were used as cutoffs for grouping according to continuous variables. Potential publication bias was assessed by the visual examination of funnel plots for symmetry, as well as the Egger regression asymmetry test [40]. The RevMan (Version 5.1; Cochrane Collaboration, Oxford, U.K.) and STATA software (Version 12.0; Stata Corporation, College Station, TX) were used for the statistical analyses.

## Results

### Results of literature search

**Figure 1:**
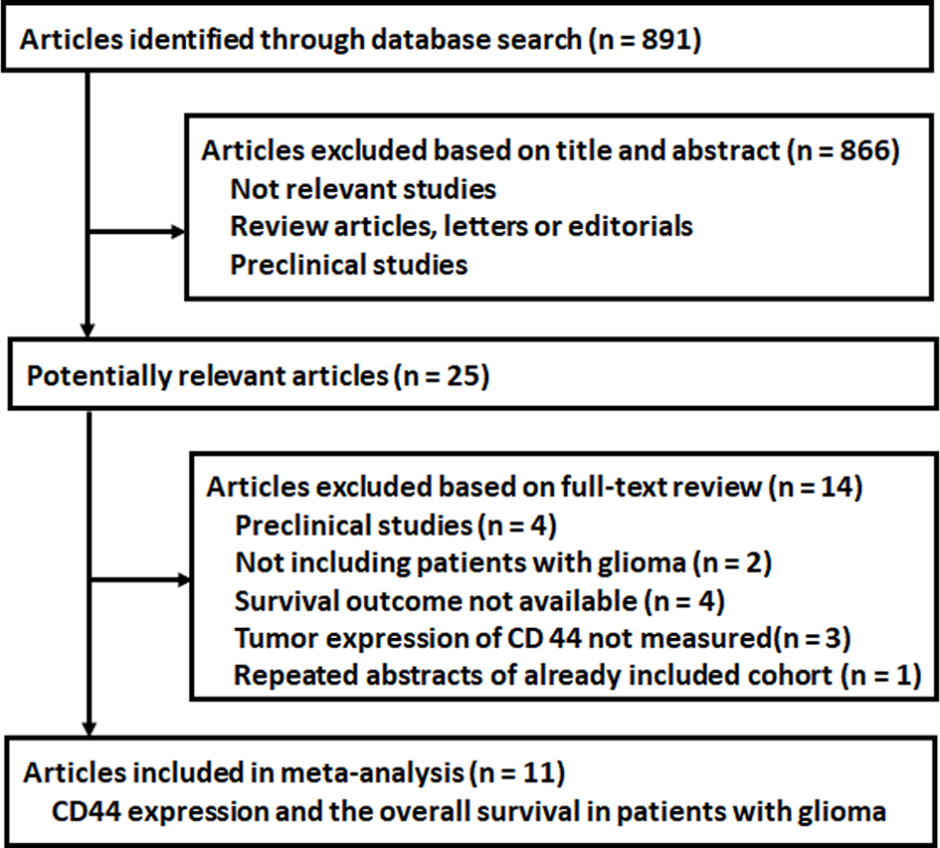
Flowchart of the database search and study identification

As shown in Figure 1, 891 studies were initially identified by the database search, and 866 were subsequently excluded based on the content of the titles and/or abstracts, mainly because they were not relevant to the purpose of this meta-analysis. The remaining 25 studies underwent full-text review. Of them, 14 studies were further excluded; four were preclinical studies, two did not include patients with glioma, four did not report the outcome of OS, three did not measure tumor expression of CD44, and one was a repeated report of an included study. Finally, 11 studies [25–35] were included for subsequent meta-analysis.

### Study characteristics and quality evaluation

The characteristics of the included studies are displayed in Table 1. Since one study reported datasets in patients with stage II–III glioma and glioblastoma independently, we included these datasets separately [26]. Overall, this meta-analysis included 967 patients with glioma from 11 retrospective cohort studies (12 datasets) performed in China [27,35], Japan [25,26], Russia [32], Spain [34], Germany [33], Sweden [29], Italy [30], France [31], and Argentina [28]. All the studies were published in English. The amount of included patients varied from 13 to 280, with mean patient ages varying from 52 to 67 years. Eight datasets included patients with glioma [25,26,30–35], while two datasets included patients with WHO grades II–III glioma [26,27], and the remaining two included patients with WHO grades II–IV glioma [28,29]. For seven studies, glioma tissue CD44 expression was measured by immunohistochemistry [28–32,34,35], while for the rest studies, quantitative reverse transcription polymerase chain reaction (qRT-PCR) was applied [25–27,33]. The median of CD44 expression values were used as cutoff for patient grouping in seven studies [25–27,31–33,35], while for the remaining four studies, CD44 expression of >70–75% was used as the cut-off value [28–30,34]. Data of HRs and 95% CI were reported in seven studies [26,27,31–35] and were estimated from survival curves in four studies [25,28–30]. Multivariate analyses adjusted for age, gender, and treatments were applied in six studies when reporting the association between CD44 level and OS [26,27,31–34], while the other five studies used univariate analyses [25,28–30,35]. The median follow-up durations varied from 15 to 40 months. The Newcastle–Ottawa scale varied from 5 to 8 in the included studies (Table 2), indicating moderate overall quality.

**Table 1:** Characteristics of the included studies

Study	Country	Study design	Sample size	Mean age	Male	WHO grade	CD44 detection method	CD44 cutoff	HR estimation	Survival analysis	Median follow-up duration	Variables adjusted
				years	%						Months	
Rauuncolo, 2002	Argentina	RC	84	52.1	61.9	II–IV	IHC	>70%	Survival curve	Univariate	40	NA
Wei, 2010	China	RC	42	NR	78.6	IV	IHC	Median	Reported	Univariate	18	NA
Sooman, 2014	Sweden	RC	97	59.5	56.7	III–IV	IHC	>75%	Survival curve	Univariate	15	NA
Guadagno, 2016	Italy	RC	25	60.3	72	IV	IHC	>70%	Survival curve	Univariate	30	NA
Pinel, 2017	France	RC	122	60.3	66.3	IV	IHC	Median	Reported	Multivariate	16	Age, gender, and treatments
Tsidulko, 2017	Russia	RC	74	52.0	52	IV	IHC	Median	Reported	Multivariate	20	Age, gender, Ki67, and treatments
Nishikawa, 2018	Japan	RC	13	NR	NR	IV	qRT-PCR	Median	Survival curve	Univariate	18	NA
Bien-Möller, 2018	Germany	RC	77	67.0	64.9	IV	qRT-PCR	Median	Reported	Multivariate	26	Age, gender, and treatments
Alameda, 2019	Spain	RC	280	59.0	58.6	IV	IHC	>70%	Reported	Multivariate	16	Age, gender, KPS, and treatments
Hou, 2019	Japan	RC	112	NR	51.8	II–III	qRT-PCR	Median	Reported	Multivariate	32	Age, gender, KPS, IDH, and treatments
Dong, 2019a	China	RC	16	NR	42.7	II–III	qRT-PCR	Median	Reported	Multivariate	36	Age, gender, and KPS
Dong, 2019b	China	RC	38	NR	61.1	IV	qRT-PCR	Median	Reported	Multivariate	36	Age, gender, and KPS

The study Dong 2019 included stratified datasets in patients with WHO grade II–III glioma and glioblastoma, which were included in the meta-analysis separately.

Abbreviations: IDH, isocitrate dehydrogenase; IHC, immunohistochemistry; KPS, Karnofsky Performance Scale; NA, not applicable; NR, not reported; RC, retrospective cohort.

**Table 2:** Details of quality evaluation by the Newcastle–Ottawa Scale

Studies	Representativeness of the exposed cohort	Selection of the non-exposed cohort	Ascertainment of exposure	Outcome of interest not present at baseline	Adjustment of age and gender	Adjustment of other confounding factors	Assessment of outcome	Follow-up long enough	Adequacy of follow-up of cohorts	Total
Rauuncolo, 2002	0	1	1	1	0	0	1	1	1	6
Wei, 2010	0	0	1	1	0	0	1	1	1	5
Sooman, 2014	0	1	1	1	0	0	1	1	1	6
Guadagno, 2016	0	1	1	1	0	0	1	1	1	6
Pinel, 2017	1	0	1	1	1	1	1	1	1	8
Tsidulko, 2017	1	0	1	1	1	1	1	1	1	8
Nishikawa, 2018	0	0	1	1	0	0	1	1	1	5
Bien-Möller, 2018	0	0	1	1	1	1	1	1	1	7
Alameda, 2019	1	0	1	1	1	1	1	1	1	8
Hou, 2019	0	0	1	1	1	1	1	1	1	7
Dong, 2019a	0	0	1	1	1	1	1	1	1	7
Dong, 2019b	0	0	1	1	1	1	1	1	1	7

The study Dong 2019 included stratified datasets in patients with WHO grade II–III glioma and glioblastoma, which were included in the meta-analysis separately.

### Expression of CD44 and OS in glioma patients

**Figure 2:**
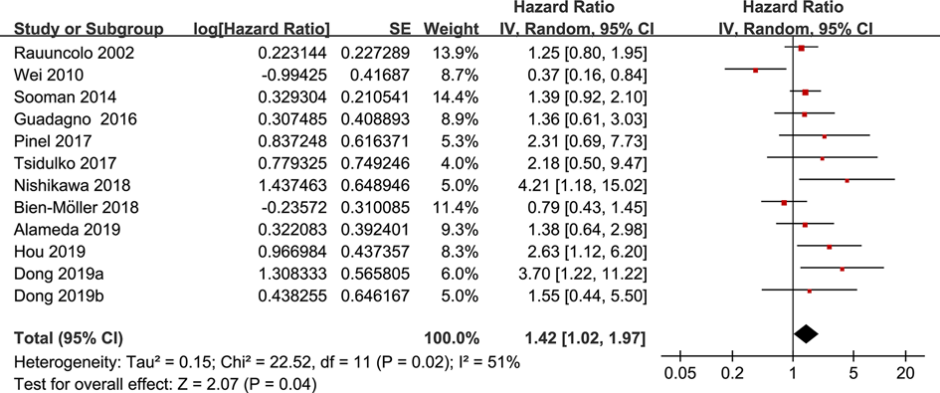
Forest plots for the meta-analysis of the prognostic efficacy of tumor CD44 for OS in patients with glioma

The pooled results of the 12 datasets from the 11 studies with a random-effect model showed that higher tumor CD44 expression significantly predicted the poor OS in patients with glioma during follow-up (HR: 1.42, 95% CI: 1.02–1.97, *P*=0.04; Figure 2) with significant heterogeneity (*P* for Cochrane’s Q test = 0.02, *I^2^* = 51%). Subgroup analyses showed that the association between tumor CD44 expression and poor OS may be different according to the WHO stages of the tumor. Specifically, higher tumor CD44 expression significantly predicted poor OS in patients with WHO stages II–III glioma (HR: 2.99, 95% CI: 1.53–5.89, *P*=0.002; *I^2^*=0%), but not in patients with glioblastoma (HR: 1.26, 95% CI: 0.76–2.08, *P*=0.47; *I^2^*=55%). The difference between the subgroups was significant (*P*=0.03; Table 3). Results were not statistically different between subgroups according to study characteristics such as patient ethnicity, sample size, CD44 detection method, CD44 cutoff, HR estimation, univariate or multivariate analysis, or median follow-up durations (*P*-values for subgroup difference all >0.10; Table 3).

**Table 3:** Subgroup analyses

	OS				
Study characteristics	Datasets number	HR (95% CI)	*I^2^*	*P* for subgroup effect	*P* for subgroup difference
**Ethnicity**					
Non-Asian	7	1.28 [1.01, 1.61]	0%	0.04	
Asian	5	1.80 [0.67, 4.81]	78%	0.24	0.50
**Sample size**					
>80	5	1.45 [1.12, 1.89]	0%	0.005	
≤80	7	1.39 [0.73, 2.64]	67%	0.32	0.89
**WHO grade**					
II–III	2	2.99 [1.52, 5.89]	0%	0.002	
IV	8	1.26 [0.76, 2.08]	55%	0.37	0.03
**CD44 detection method**					
IHC	6	1.21 [0.75, 1.95]	52%	0.45	
qRT-PCR	6	1.72 [1.03, 2.89]	57%	0.04	0.32
**CD44 cutoff**					
IHC	8	1.63 [0.86, 3.08]	69%	0.14	
qRT-PCR	4	1.33 [1.02, 1.74]	0%	0.03	0.57
**HR estimation**					
Survival curve	4	1.41 [1.06, 1.87]	4%	0.02	
Reported	8	1.41 [0.81, 2.46]	63%	0.22	0.99
**Survival analysis**					
Multivariate	7	1.68 [1.06, 2.64]	35%	0.03	
Univariate	5	1.20 [0.72, 2.01]	67%	0.49	0.34
**Median follow-up duration**					
>20 months	6	1.46 [0.97, 2.20]	42%	0.07	
≤20 months	6	1.40 [0.77, 2.54]	64%	0.28	0.90

### Publication bias

**Figure 3:**
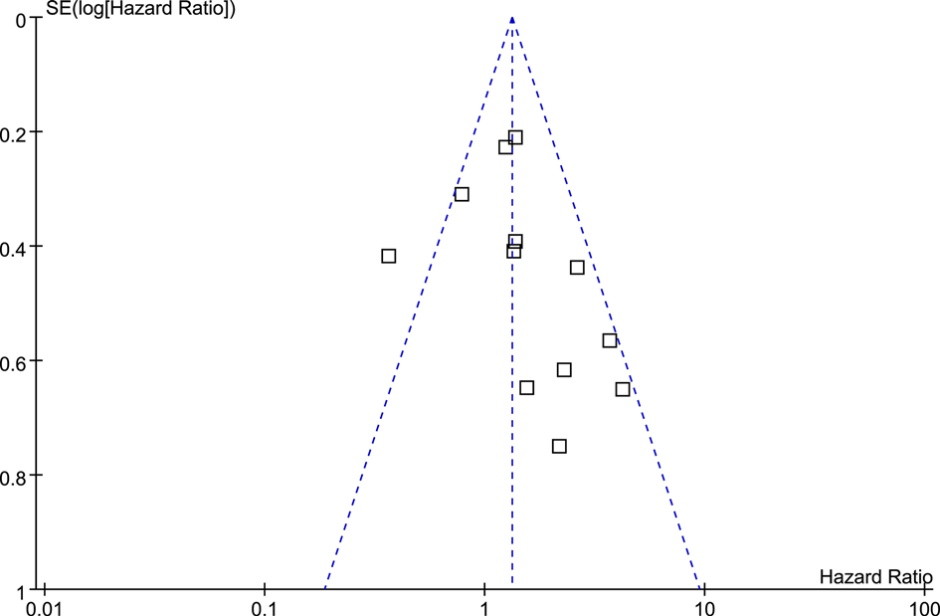
Funnel plots for the meta-analysis of the prognostic efficacy of tumor CD44 for OS in patients with glioma

The funnel plots for the meta-analysis of the prognostic efficacy of tumor CD44 level for poor OS are shown in Figure 3. The funnel plots were determined to be symmetrical based on visual inspection, suggesting low risk of publication bias. The result of the Egger’s regression test for the meta-analysis also demonstrated similar result (*P*=0.37).

## Discussion

In the present study, by pooling the results of available cohort studies, we found that higher tumor expression of CD44 is a prognostic factor for poor survival in patients with glioma. Subsequent subgroup analyses showed that disease stages may affect the potential prognostic efficacy of tumor CD44 expression for the survival in patients with glioma. Specifically, the predictive efficacy of higher CD44 tumor expression for poor OS may be significant in patients with WHO stage II–III glioma, but not for those with glioblastoma (WHO stage IV). In addition, the association between higher expression of CD44 and poor survival in patients with glioma does not seem to affect by study characteristics such as patient ethnicity, sample size, CD44 detection method, CD44 cutoff, HR estimation strategy, univariate or multivariate analysis, or median follow-up durations. Taken together, these results demonstrated that higher tumor expression of CD44 may predict poor survival in patients with glioma, particularly in those with WHO stage II–III glioma. These findings should be validated in large-scale prospective cohort studies, and the clinical importance of tumor CD44 for the risk stratification and treatment of glioma deserve further investigation.

To the best of our knowledge, this is the first meta-analysis to evaluate the predictive value of higher tumor CD44 expression for the prognosis in patients with glioma. The potential association between higher tumor expression of CD44 and overall poor prognosis in patients with glioma could be explained by the roles of CD44 in the proliferation, invasion, metastasis of glioma, and resistance to chemotherapy and radiation therapy [24]. CD44, as a cell membrane glycoprotein, exerts diverse cellular processes including cell motility, proliferation, apoptosis, and angiogenesis, via binding extracellular ligands, principally hyaluronic acid (HA). Increased expression of CD44 has been early confirmed in human glioma cells as compared with normal brain tissue, and suppression of CD44 expression decreases migration and invasion of human glioma cells [41]. In another study, depletion of CD44 was shown to block glioblastoma growth and sensitizes glioblastoma cells to cytotoxic drugs *in vivo*, and CD44 functions upstream of the mammalian Hippo signaling pathway in glioblastoma cells [42]. Moreover, tumor expression of CD44 is shown to be up-regulated during glioma progression in the brain, which may aid in tracing and targeting the invading glioma cells [43]. Importantly, a subsequent study showed that CD44 transduces HA-based stiffness cues, temporally precedes integrin-based adhesion maturation, and facilitates invasion, demonstrating that the HA-CD44 axis is critical for cell adhesion, invasion, and mechanosensing of an HA-based matrix in glioma [44]. Stemness of cancer cells has been associated with poor survival [45]. It was shown that CD44 modulates the hypoxic response of glioma cells and that the pseudo-hypoxic phenotype of stem-like glioma cells is achieved by stabilization of hypoxia-inducible factors-2α through interaction with CD44, thereby contributing to the stemness of glioma cells [46]. These studies highlight the multiple roles of CD44 in the pathogenesis and invasion of glioma, and the potential therapeutic significance of CD44 depletion for patients with glioma. Further studies are needed to determine the key signaling pathways underlying the role of CD44 in glioma, which may be helpful to develop the CD44-based target therapy for patients with glioma.

Our subgroup analysis showed that the predictive efficacy of higher CD44 tumor expression for poor OS may be significant in patients with WHO stage II–III glioma, but not for those with glioblastoma. The potential mechanisms underlying these findings remain unclear. However, from a clinical perspective, since the median survival in patients with WHO stage II–III glioma is generally longer than those with glioblastoma, introducing additional risk stratification factors besides WHO grade system may be more important in those with stage II–III glioma as compared with patients with glioblastoma. A previous study showed that the expression of CD44 in glioblastoma may be heterogeneous, and the tumorigenicity of primary GBM differs between CD44^low^/CD133^high^ and CD44^high^/CD^133^low for gene expression profiles [47]. Subsequently, the molecular heterogeneity among tumors may affect the prognostic value of CD44 in glioblastoma [25]. The exact mechanisms underlying the potential difference between the prognostic power of higher tumor CD44 expression for survival in patients with WHO stage II–III glioma and in those with glioblastoma deserve further investigation.

The strengths of our study include extensive literature search, rigorous meta-analysis according to the MOOSE and Cochrane’s guidelines, and comprehensive subgroup analyses. However, our study has the following limitations which should be noted when interpreting the results. First, only 11 studies with 12 datasets were included. The number of available studies remains small. Therefore, results of subgroup analyses should be interpreted with caution. Large-scale cohort studies are needed to confirm our results. Second, only retrospective cohort studies were included in the meta-analysis, which may be confounded by limitations such as recall bias. Therefore, prospective studies are needed to validate these findings. Third, significant heterogeneity existed among the included studies. Although subgroup analyses showed that WHO stages of the diseases might contribute to the heterogeneity, results of subgroup analyses should be treated cautiously since the limited studies available for each stratum. Besides, other factors, such as comorbidities of the patients, cancer treatments, and cut-off value to determine the increased tumor CD44 expression may also contribute to the heterogeneity. However, since these factors were rarely reported in each study, we were unable to evaluate the potential prognostic role of tumor CD44 expression for survival according to these characteristics. Future studies are warranted. In addition, since the cut-off values for grouping patients with higher or lower tumor CD44 varied among the studies, it may lead to heterogeneity. The optimal tumor CD44 cut-off values for the prognosis prediction in patients with glioma should be determined in future studies. Finally, the potential therapeutic significance of CD44 as a treatment target for glioma also should be determined in the future.

In conclusion, results of meta-analysis showed that higher tumor expression of CD44 may predict poor survival in patients with glioma, particularly in those with WHO stage II–III glioma. These findings should be validated in large-scale prospective cohort studies, and the clinical role of CD44 for glioma deserves further investigation.

## Abbreviations

CI: confidence interval
CSC: cancer stem cell
HA: hyaluronic acid
HR: hazard ratio
OS: overall survival
WHO: World Health Organization

## References

[1] Lapointe S., Perry A. and Butowski N.A. (2018) Primary brain tumours in adults. Lancet 392, 432–446 10.1016/S0140-6736(18)30990-5 30060998

[2] Bates A., Gonzalez-Viana E., Cruickshank G. and Roques T. (2018) Primary and metastatic brain tumours in adults: summary of NICE guidance. BMJ 362, k2924 10.1136/bmj.k2924 30018066

[3] Brandner S. and Jaunmuktane Z. (2018) Neurological update: gliomas and other primary brain tumours in adults. J. Neurol. 265, 717–727 10.1007/s00415-017-8652-3 29098416 PMC5834564

[4] Gupta A. and Dwivedi T. (2017) A Simplified Overview of World Health Organization Classification Update of Central Nervous System Tumors 2016. J. Neurosci. Rural Pract. 8, 629–641 29204027 10.4103/jnrp.jnrp_168_17 PMC5709890

[5] Louis D.N., Perry A., Reifenberger G. et al. (2016) The 2016 World Health Organization Classification of Tumors of the Central Nervous System: a summary. Acta Neuropathol. 131, 803–820 10.1007/s00401-016-1545-1 27157931

[6] Quan G., Zheng Y., Chen J. et al. (2018) Prediction value of unmeasurable MR enhancement at early stage after gross-total resection on the survival state of patients with high-grade glioma. J. Neuro Oncol. 140, 359–366 10.1007/s11060-018-2961-y 30182160

[7] Wang Z.L., Zhang C.B., Liu Y.Q., Wang Z. and Jiang T. (2019) Peripheral blood test provides a practical method for glioma evaluation and prognosis prediction. CNS Neurosci.Ther. 25, 876–883 10.1111/cns.13120 PMC6630006 30912613

[8] Yang L., Shi P., Zhao G. et al. (2020) Targeting cancer stem cell pathways for cancer therapy. Signal Transduct. Target Ther. 5, 8 10.1038/s41392-020-0110-5 32296030 PMC7005297

[9] Budillon A., Curley S., Fusco R. and Mancini R. (2019) Identification and targeting of stem cell-activated pathways in cancer therapy. Stem Cells Int. 2019, 8549020 10.1155/2019/8549020 31281388 PMC6589219

[10] Vermeulen L., de Sousa e Melo F., Richel D.J. and Medema J.P. (2012) The developing cancer stem-cell model: clinical challenges and opportunities. Lancet Oncol. 13, e83–89 10.1016/S1470-2045(11)70257-1 22300863

[11] Najafi M., Mortezaee K. and Majidpoor J. (2019) Cancer stem cell (CSC) resistance drivers. Life Sci. 234, 116781 10.1016/j.lfs.2019.116781 31430455

[12] Murar M. and Vaidya A. (2015) Cancer stem cell markers: premises and prospects. Biomark. Med. 9, 1331–1342 10.2217/bmm.15.85 26612591

[13] Kim W.T. and Ryu C.J. (2017) Cancer stem cell surface markers on normal stem cells. BMB Rep. 50, 285–298 10.5483/BMBRep.2017.50.6.039 28270302 PMC5498139

[14] Skandalis S.S., Karalis T.T., Chatzopoulos A. and Karamanos N.K. (2019) Hyaluronan-CD44 axis orchestrates cancer stem cell functions. Cell. Signal. 63, 109377 10.1016/j.cellsig.2019.109377 31362044

[15] Zhao S., He J.L., Qiu Z.X. et al. (2014) Prognostic value of CD44 variant exon 6 expression in non-small cell lung cancer: a meta-analysis. Asian Pac. J. Cancer Prev. 15, 6761–6766 10.7314/APJCP.2014.15.16.6761 25169522

[16] Chen Y., Fu Z., Xu S., Xu Y. and Xu P. (2014) The prognostic value of CD44 expression in gastric cancer: a meta-analysis. Biomed. Pharmacother. 68, 693–697 10.1016/j.biopha.2014.08.001 25194445

[17] Luo Y. and Tan Y. (2016) Prognostic value of CD44 expression in patients with hepatocellular carcinoma: meta-analysis. Cancer Cell Int. 16, 47 10.1186/s12935-016-0325-2 27330410 PMC4912706

[18] Wang Z., Tang Y., Xie L. et al. (2019) The prognostic and clinical value of CD44 in colorectal cancer: a meta-analysis. Front. Oncol. 9, 309 10.3389/fonc.2019.00309 31114754 PMC6503057

[19] Li X., Ma X., Chen L. et al. (2015) Prognostic value of CD44 expression in renal cell carcinoma: a systematic review and meta-analysis. Sci. Rep. 5, 13157 10.1038/srep13157 26287771 PMC4541415

[20] Wang Z., Wang Q., Wang Y. and Chen J. (2017) Prognostic significance of CD24 and CD44 in breast cancer: a meta-analysis. Int. J. Biol. Markers 32, e75–e82 10.5301/jbm.5000224 27470135

[21] Lin J. and Ding D. (2017) The prognostic role of the cancer stem cell marker CD44 in ovarian cancer: a meta-analysis. Cancer Cell Int. 17, 8 10.1186/s12935-016-0376-4 28070170 PMC5216581

[22] Chen J., Zhou J., Lu J., Xiong H., Shi X. and Gong L. (2014) Significance of CD44 expression in head and neck cancer: a systemic review and meta-analysis. BMC Cancer 14, 15 10.1186/1471-2407-14-15 24410905 PMC3893437

[23] Zhang Y., Ding C., Wang J. et al. (2015) Prognostic significance of CD44V6 expression in osteosarcoma: a meta-analysis. J. Orthop. Surg. Res. 10, 187 10.1186/s13018-015-0328-z 26697855 PMC4690422

[24] Mooney K.L., Choy W., Sidhu S. et al. (2016) The role of CD44 in glioblastoma multiforme. J. Clin. Neurosci. 34, 1–5 10.1016/j.jocn.2016.05.012 27578526

[25] Nishikawa M., Inoue A., Ohnishi T. et al. (2018) Significance of glioma stem-like cells in the tumor periphery that express high levels of CD44 in tumor invasion, early progression, and poor prognosis in glioblastoma. Stem Cells Int. 2018, 5387041 10.1155/2018/5387041 30210550 PMC6126065

[26] Dong Q., Li Q., Wang M. et al. (2019) Elevated CD44 expression predicts poor prognosis in patients with low-grade glioma. Oncol. Lett. 18, 3698–3704 31516582 10.3892/ol.2019.10728 PMC6732950

[27] Hou C., Ishi Y., Motegi H. et al. (2019) Overexpression of CD44 is associated with a poor prognosis in grade II/III gliomas. J. Neuro Oncol. 145, 201–210 10.1007/s11060-019-03288-8 31506754

[28] Ranuncolo S.M., Ladeda V., Specterman S. et al. (2002) CD44 expression in human gliomas. J. Surg. Oncol. 79, 30–35, 10.1002/jso.10045 11754374

[29] Sooman L., Freyhult E., Jaiswal A. et al. (2015) FGF2 as a potential prognostic biomarker for proneural glioma patients. Acta Oncol. 54, 385–394 10.3109/0284186X.2014.951492 25263081

[30] Guadagno E., Borrelli G., Califano M., Cali G., Solari D. and Del Basso De Caro M. (2016) Immunohistochemical expression of stem cell markers CD44 and nestin in glioblastomas: evaluation of their prognostic significance. Pathol. Res. Pract. 212, 825–832 10.1016/j.prp.2016.07.002 27450656

[31] Pinel B., Duchesne M., Godet J. et al. (2017) Mesenchymal subtype of glioblastomas with high DNA-PKcs expression is associated with better response to radiotherapy and temozolomide. J. Neuro Oncol. 132, 287–294 10.1007/s11060-016-2367-7 28070830

[32] Tsidulko A.Y., Kazanskaya G.M., Kostromskaya D.V. et al. (2017) Prognostic relevance of NG2/CSPG4, CD44 and Ki-67 in patients with glioblastoma. Tumour Biol. 39, 1010428317724282 10.1177/1010428317724282 28945172

[33] Bien-Moller S., Balz E., Herzog S. et al. (2018) Association of glioblastoma multiforme stem cell characteristics, differentiation, and microglia marker genes with patient survival. Stem Cells Int. 2018, 9628289 10.1155/2018/9628289 29535786 PMC5822829

[34] Alameda F., Velarde J.M., Carrato C. et al. (2019) Prognostic value of stem cell markers in glioblastoma. Biomarkers 24, 677–683 10.1080/1354750X.2019.1652345 31496301

[35] Wei K.C., Huang C.Y., Chen P.Y. et al. (2010) Evaluation of the prognostic value of CD44 in glioblastoma multiforme. Anticancer Res. 30, 253–259 20150644

[36] Stroup D.F., Berlin J.A., Morton S.C. et al. (2000) Meta-analysis of observational studies in epidemiology: a proposal for reporting. Meta-analysis Of Observational Studies in Epidemiology (MOOSE) group. JAMA 283, 2008–2012 10.1001/jama.283.15.2008 10789670

[37] Higgins J. and Green S. (2011) Cochrane Handbook for Systematic Reviews of Interventions Version 5.1.0. The Cochrane Collaboration www.cochranehandbook.org

[38] Wells G.A., Shea B., O’Connell D. et al. (2010) The Newcastle-Ottawa Scale (NOS) for assessing the quality of nonrandomised studies in meta-analyses. http://www.ohri.ca/programs/clinical_epidemiology/oxford.asp

[39] Higgins J.P. and Thompson S.G. (2002) Quantifying heterogeneity in a meta-analysis. Stat. Med. 21, 1539–1558 10.1002/sim.1186 12111919

[40] Egger M., Davey Smith G., Schneider M. and Minder C. (1997) Bias in meta-analysis detected by a simple, graphical test. BMJ 315, 629–634 10.1136/bmj.315.7109.629 9310563 PMC2127453

[41] Okada H., Yoshida J., Sokabe M., Wakabayashi T. and Hagiwara M. (1996) Suppression of CD44 expression decreases migration and invasion of human glioma cells. Int. J. Cancer 66, 255–260 10.1002/(SICI)1097-0215(19960410)66:2<255::AID-IJC20>3.0.CO;2-A 8603821

[42] Xu Y., Stamenkovic I. and Yu Q. (2010) CD44 attenuates activation of the hippo signaling pathway and is a prime therapeutic target for glioblastoma. Cancer Res. 70, 2455–2464 10.1158/0008-5472.CAN-09-2505 20197461 PMC2840073

[43] Wiranowska M., Ladd S., Smith S.R. and Gottschall P.E. (2006) CD44 adhesion molecule and neuro-glial proteoglycan NG2 as invasive markers of glioma. Brain Cell Biol. 35, 159–172 10.1007/s11068-007-9009-0 17957481

[44] Kim Y. and Kumar S. (2014) CD44-mediated adhesion to hyaluronic acid contributes to mechanosensing and invasive motility. Mol. Cancer Res. 12, 1416–1429 10.1158/1541-7786.MCR-13-0629 24962319 PMC4201971

[45] Saygin C., Matei D., Majeti R., Reizes O. and Lathia J.D. (2019) Targeting cancer stemness in the clinic: from hype to hope. Cell Stem Cell 24, 25–40 10.1016/j.stem.2018.11.017 30595497

[46] Johansson E., Grassi E.S., Pantazopoulou V. et al. (2017) CD44 interacts with HIF-2alpha to modulate the hypoxic phenotype of perinecrotic and perivascular glioma cells. Cell Rep. 20, 1641–1653 10.1016/j.celrep.2017.07.049 28813675

[47] Fu J., Yang Q.Y., Sai K. et al. (2013) TGM2 inhibition attenuates ID1 expression in CD44-high glioma-initiating cells. Neuro Oncol. 15, 1353–1365 10.1093/neuonc/not079 23877317 PMC3779037

